# Management of Delayed Onset Postoperative Hemorrhage after Anastomotic Urethroplasty

**DOI:** 10.1155/2015/646784

**Published:** 2015-11-09

**Authors:** L. A. Bertrand, S. P. Elliott, B. N. Breyer, B. A. Erickson

**Affiliations:** ^1^Department of Urology, University of Iowa Hospitals and Clinics, 200 Hawkins Drive, 3RCP, Iowa City, IA 52242, USA; ^2^Department of Urology, University of Minnesota, 420 Delaware Street, SE b435, Minneapolis, MN 55455, USA; ^3^Department of Urology, University of California San Francisco, 1001 Potrero Avenue, SFGH 3, San Francisco, CA 94110, USA

## Abstract

Excision with primary anastomosis (EPA) urethroplasty is generally the preferred method for short strictures in the bulbar urethra, given its high success rate and low complication rate compared to other surgical interventions. Bleeding is a presumed risk factor for any surgical procedure but perioperative hemorrhage after an EPA requiring hospitalization and/or reintervention is unreported with no known consensus on the best course for management. Through our experience with three separate cases of significant postoperative urethral hemorrhage after EPA, we developed an algorithm for treatment beginning with conservative management and progressing through endoscopic and open techniques, as well as consideration of embolization by interventional radiology. All the three of these cases were managed successfully though they did require multiple interventions. We theorize that younger patients with more robust corpus spongiosum and more vigorous spontaneous erections, patients that have undergone fewer prior urethral procedures and therefore have more prominent vasculature, and those patients managed with a two-layer closure of the ventral urethra without ligation of the transected bulbar arteries are at a higher risk for this complication.

## 1. Introduction

Urethral reconstruction is the gold standard for treatment of anterior urethral stricture disease. For short strictures in the bulbar urethra, excision with primary anastomosis (EPA) is generally the preferred method given its high success rate and low complication rate when compared to other endoscopic or open reconstructive techniques. Complications with EPA are rare [1, 2] but can include infection, erectile dysfunction [3, 4], wound breakdown, and recurrence of the urethral stricture [5]. Perioperative bleeding requiring hospitalization and/or reintervention is a presumed risk but is unreported. Postoperative urethral hemorrhage can be extremely challenging to manage as it generally involves bleeding from the transected bulbar arteries. To our knowledge, there is no consensus on the best way to manage such a patient.

The purpose of this report is to discuss the presentation and management of three patients from three different institutions in which postoperative urethral hemorrhage after EPA led to the need for rehospitalization. Here, we will discuss the various methods used for management.

## 2. Case Presentation

### 2.1. Patient #1

Our first patient was a 32-year-old diabetic male with a body mass index (BMI) of 36 kg/m^2^ and a 2-centimeter bulbar urethral stricture managed with a traditional EPA on Christmas Eve. In summary, after excision of the strictured urethra, a spatulated anastomosis was performed: the dorsal anastomosis is a single layer of interrupted 5-0 PDS (i.e., includes both sponge and mucosa) and the ventral anastomosis is in two interrupted layers (i.e., separate mucosal and adventitial/sponge closures) and a watertight closure is achieved. His procedure was uncomplicated, although some inflammatory changes were noted on the dorsal wall of the 28-French (Fr) distal urethral stump. The patient was discharged home the following day with a catheter in place. He represented on postoperative day (POD) #12 with brisk, bright red urethral bleeding around the catheter. There was only mild swelling in the perineum and scrotum and no butterfly sign indicating significant hematoma. We applied manual compression to the perineum for 30 minutes that improved bleeding temporarily. However, with ambulation, the bleeding resumed and it was then decided to proceed with reexploration of the surgical wound. A 1,000 cc bag of intravenous saline was placed under patient's perineum with patient in an upright position until the surgery could be performed a couple of hours later.

Under anesthesia the urethral catheter was removed and cystoscopy revealed a partial disruption of the dorsal wall of the anastomosis. There was not obvious area of the urethra that could be fulgurated with electrocautery and thus the perineal incision was reopened revealing no perineal hematoma. The urethra was then rotated 180 degrees to reveal the dorsal wall; the disruption was appreciated and bleeding was seen from the exposed spongiosum. The remaining dorsal wall stitches in the area of the disruption were excised, revealing the underlying urethral mucosa which looked inflamed. We placed several interrupted anastomotic stitches of 6-0 PDS in the urethral mucosa in the area of the disruption and then ran the outer wall of the spongiosum with 5-0 PDS successfully stopping the bleeding. Operative cystoscopy was repeated and confirmed no bleeding and an intact dorsal wall.

The catheter was removed 3 weeks later and a VCUG showed no leakage of contrast. At the 3-month postoperative visit, cystoscopy confirmed a 28-Fr lumen and uroflowmetry revealed a maximum flow rate of 42 mL/s.

### 2.2. Patient #2

Patient #2 is an 18-year-old male with a 2 cm bulbar urethral stricture which was managed with an EPA urethroplasty as previously described. His catheter was removed on POD #14. His voiding cystourethrogram showed a very small area of extravasation at the level of the anastomosis. On POD #21, the patient began having brisk bleeding around the catheter and thus returned to the hospital. The bleeding would subside with perineal pressure and patient was able to be discharged. Over the next 4–6 weeks, however, patient returned multiple times with continued, intermittent bleeding around this catheter. He required a transfusion of 2 units of blood. Given multiple recurrences, he was eventually brought to the operating room for endoscopic management. A cystoscope was advanced into the urethra to the level of the anastomosis in the bulbar urethra. A briskly bleeding vessel was encountered at a site of mucosal disruption at the 5 o'clock position (ventral) which was fulgurated with an electrocautery probe (Bugbee). The bleeding ceased and a urethral catheter was placed. Patient initially did well but about one week later again presented with brisk bleeding around his catheter. He was again taken to the operating room for another endoscopic procedure. The electrocautery probe (Bugbee) was again used to fully fulgurate the hemorrhaging tissues. The patient subsequently did well and suffered no further bleeding. He remained recurrence-free at 14 months.

### 2.3. Patient #3

Patient #3 is a 17-year-old otherwise healthy male with history of obstructive urinary symptoms who was found to have a 1 cm proximal bulbar urethral stricture on retrograde urethrogram. The patient underwent an uncomplicated EPA repair, with technique similar to that described above, with the total operative time of 2.5 hours and total blood loss of 150 cc. In this case, the patient was discharged home on POD #1 with a 16-Fr catheter in place.

The patient returned on POD #8 when he began to have bright red blood around the catheter. The bleeding had been ongoing for nearly 4 hours by the time he reached the emergency room and his hemoglobin (Hgb) dropped from a value of 10.3 upon initial arrival to the emergency room to 7.5 at recheck a couple of hours later. A pressure dressing was applied to the perineum using rolled Kerlix bandages and ACE bandages that acted as a sling and applied direct pressure to the surgical site. The dressing remained in place for 12 hours and was then removed revealing no active bleeding. He was discharged home with a catheter in place and stabilized Hgb of 7.8.

**Figure 1:**
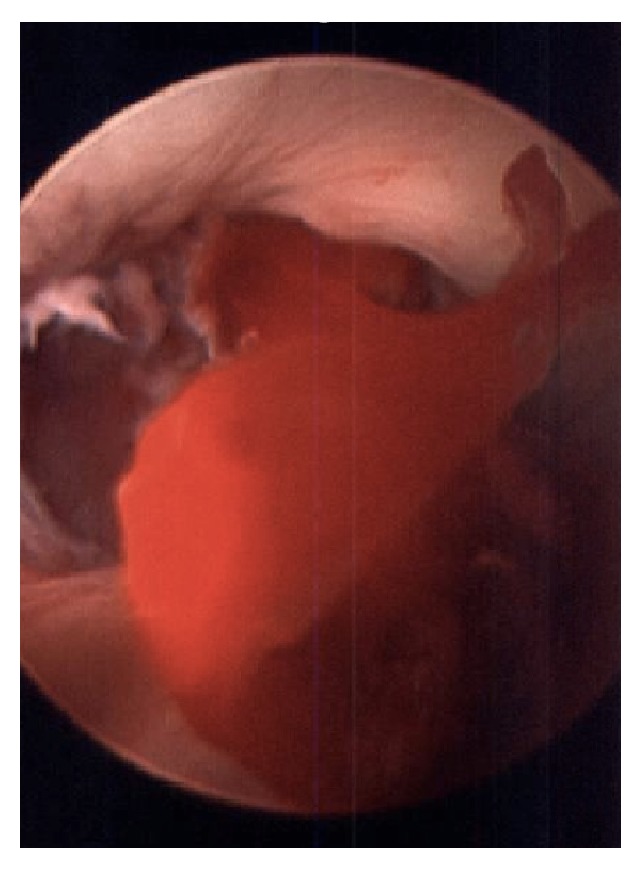
Intraurethral arterial bleeding.

**Figure 2:**
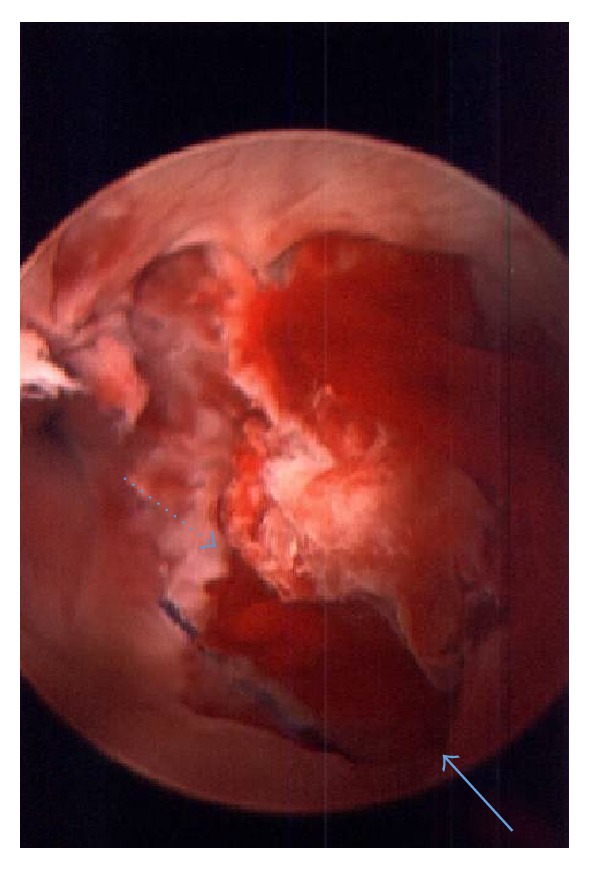
Ventral anastomotic disruption; distal end (solid arrow) and proximal end (dotted arrow) of anastomosis.

The patient returned on POD #10 with recurrent brisk bleeding that began after a bowel movement. Upon examination in the emergency room, arterial bleeding was again noted to be coming from around the urethral catheter. The perineum remained soft. Two units of packed red blood cells were provided in the emergency room after the Hgb was noted to be 6.8 and the patient was brought semiemergently to the operating room for a cystoscopic evaluation. A 22-Fr rigid cystoscope was inserted into the urethra and advanced to the level of the urethroplasty anastomosis where a large clot was visualized with active bleeding (Figure 1). An electrocautery (Bugbee) probe was first used to manipulate the clot loose and pulsatile bleeding was seen from roughly the 5 o'clock position of the anastomosis where the ventral mucosal edges were seen to have pulled apart (Figure 2). An attempt at fulguration of the vessel, with care not to fulgurate the entire bulbar urethra, using the Bugbee was unsuccessful. Next, fibrin glue (lyophilized pooled human concentrate fibrinogen plus bovine thrombin) was then injected through a long (laparoscopic) applicator under direct visualization into the area of the mucosal opening. After a total of 8 cc of fibrin glue was injected, the bleeding stopped. He was discharged from the hospital 2 days later with a 16 F catheter in place.

The patient then returned on POD #19 when brisk urethral bleeding was again noted. Pressure dressing was successful in stopping the bleeding, but it recurred immediately after removal of the dressing. We discussed two options at this point with the patient and family: (1) interventional radiology embolization and (2) open perineal exploration. The patient and family elected open exploration. The prior incision was utilized and the urethra was identified. There was no appreciable periurethral bleeding or hematoma. The ventral adventitial anastomosis was then opened and the mucosal disruption was immediately noted to be adjacent to an actively bleeding bulbar artery. The artery was ligated with a 4-0 Vicryl and a single-layer ventral anastomosis with a 4-0 Vicryl was then performed as the two layers were now indistinguishable. The patient was discharged on POD #21 (POD #2 from open repair) with stable Hgb; he did not return with further bleeding episodes. The catheter was removed 2 weeks following the second perineal exploration after a pericatheter retrograde urethrogram revealed no leak. The patient remains stricture-free (confirmed on cystoscopy) at his 12-month follow-up and he has no postoperative voiding complaints or erectile dysfunction.

## 3. Discussion

The purpose of this was study was to review three cases of postoperative urethral bleeding after uncomplicated excision and primary anastomotic urethroplasty so as to provide clinically useful case material for future urethroplasty bleeding episodes. Reported rates of any bleeding after urethroplasty are 0–2.6% [1, 2], but to our knowledge, the rates and management strategies for clinically significant postoperative urethral bleeding requiring intervention have not been reported previously.

### 3.1. Proposed Mechanism

The average age of the three patients presented here was 22. We do not believe this young age is coincidental and hypothesize that because younger men often have larger, more robust corpora spongiosum and generally have undergone fewer prior urethrotomies (relative to older stricture patients), they have more prominent vasculature and thus are more likely to have bleeding from their bulbar urethras postoperatively. Younger patients are also more likely to achieve frequent and vigorous spontaneous erections, which may place additional tension on the fresh anastomosis, possibly leading to mucosal disruption and exposure of the bulbar vessels.

The technique performed in these three patients might also be contributory. All three cases were managed with a two-layer closure of the ventral urethra without ligation of the transected bulbar artery. In all three cases, the source of bleeding was positively identified at the time of either endoscopic or open management, and in two of the three cases, the bleeding was noted to be coming from the ventral urethra where this two-layer closure was performed. The bulbar arteries are purposely not ligated during these procedures with the hope that these arteries will ultimately reconstitute and continue to provide the distal urethra with fresh oxygenated blood. However, while this reconstitution is an accepted theory, it has not been proven. In all three cases above, an artery was likely incorporated in a closure stitch leading to relatively late presenting arteriourethral fistula formation and pulsatile bleeding.

### 3.2. Proposed Management Algorithm

The mainstay of treatment should be direct pressure on the perineum to compress this area of the bulbar urethra. In all three cases described above, this treatment at least temporarily controlled the bleeding and presumably decreased overall blood loss. However, we believe this method must be employed with caution as prolonged pressure to the perineum, especially in the setting of a fresh anastomosis, can lead to local ischemia compromising the anastomosis and/or penile blood flow. In addition, we believe the material used for compression should be chosen carefully. Here we describe the use of gauze and saline bags, both of which are compressible. Less compressible materials, such as rolls of tape, hard plastics, woods, or metals, may increase the risk of ischemia and should be avoided.

Endoscopic management was utilized in two of the three cases above and successfully stopped bleeding for one week in case #3 and permanently in case #2. In both cases, a ventral mucosal disruption was noted to demonstrate arterial bleeding. Electrocautery (Bugbee) fulguration was successful at least temporarily in both cases and provided permanent solution in case #2. While the temptation exists for extensive fulguration of the area in order to ensure full hemostasis, care should be taken not to burn too comprehensively as this could compromise the anastomosis; it is important to balance this risk with the risk of rebleeding. We recommend spot fulguration in short bursts with frequent reassessments of the anastomosis and keeping the cautery intensity as low as possible while still achieving coagulation.

Open exploration was utilized in cases #1 and #3 after unsuccessful attempts at conservative management. While reopening a surgical perineal wound is never ideal, in these cases it was necessary after other conservative measures failed. Importantly, at nearly 2 weeks after the original surgery, little difficulty was encountered with adhesions or tissue planes in either case. Closure that incorporated the bleeding bulbar artery was performed and resulted in good hemostasis and good long-term outcomes after the procedures with no restricturing of either patient's urethra at 12 months out from surgery.

In all cases, interventional radiologic embolization was considered but not attempted. The primary concerns were the inability to selectively embolize the small bulbar branch from the internal pudendal artery (via the penile artery), the potential for further local anastomotic ischemia, and the potential for inadvertent embolization of the cavernosal arteries (also a branch of the internal pudendal artery), which could lead to erectile dysfunction. Still, while embolization was not attempted, embolization of these small arteries has been described before. The trauma literature recognizes embolization as a viable option for management of bulbar area hemorrhage in cases of trauma to the urethra [6]. One case describes a bulbar urethral injury, which presented with delayed, uncontrolled urethral hemorrhage. Angiography was done which showed a pseudoaneurysm arising from the left bulbourethral artery with active urethral extravasation. Coil embolization successfully managed the bleeding without de novo erectile dysfunction. A second case report describes embolization as treatment for delayed postoperative urethral bleeding after an open radical prostatectomy for prostate cancer [7]. On CT angiography, the patient was found to have active bleeding at a bulbar artery arising from the left internal pudendal artery without associated pelvic hematoma. Selective embolization was successfully performed with absorbable material.

## 4. Conclusion

**Figure 3:**
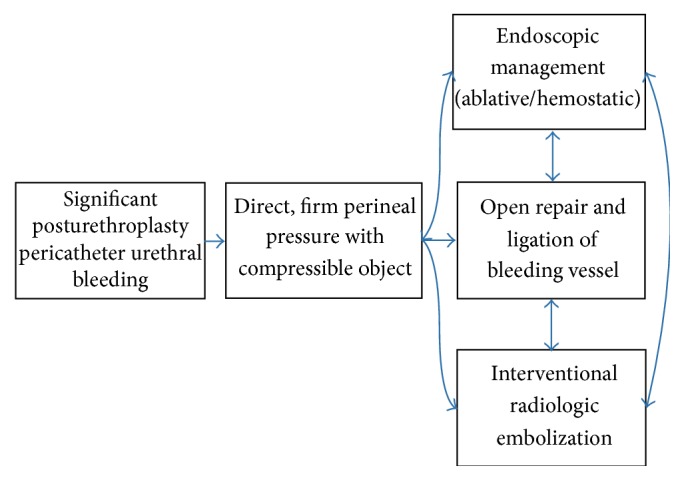
Treatment algorithm.

Although significant urethral bleeding after urethral reconstruction is rare, it can be associated with substantial morbidity and should be dealt with promptly. We present a series of three cases and propose a management algorithm (Figure 3). Direct pressure to the surgical wound should be applied initially and will stop most bleeding, at least temporarily. Secondary management can include endoscopic techniques that are both ablative and hemostatic. Finally, with bleeding recalcitrant to conservative measures, surgical exploration provides direct access to the bleeding vessels (generally the transected bulbar arteries) which are easily ligated. Embolization is an option that was considered but not attempted in these three cases, though it is likely safe and effective in appropriate situations.
